# A Systematic Review of Fibromyalgia and Recent Advancements in Treatment: Is Medicinal Cannabis a New Hope?

**DOI:** 10.7759/cureus.17332

**Published:** 2021-08-20

**Authors:** Hajra Khurshid, Israa A Qureshi, Nasrin Jahan, Terry R Went, Waleed Sultan, Alisha Sapkota, Michael Alfonso

**Affiliations:** 1 Medicine, California Institute of Behavioral Neurosciences & Psychology, Fairfield, USA; 2 Psychiatry, California Institute of Behavioral Neurosciences & Psychology, Fairfield, USA

**Keywords:** fibromyalgia, chronic pain, cannabis, cannabinoids, pharmacotherapy, alternative therapy

## Abstract

Fibromyalgia syndrome (FMS) is a pain disorder characterized by chronic widespread pain, fatigue, and sleep disturbance, in the absence of any well-defined underlying organic disease. The exact pathophysiology and the mechanism which links different factors related to the disease is still unknown. Due to unknown precise pathogenesis, the coexistence of other diseases, and overlapping clinical features, FMS diagnosis may be laborious. Various treatment strategies are used, only a few Food and Drug Administration (FDA) approved, still we are facing challenges regarding effective treatment. Recently, medicinal cannabis has proven to be effective in chronic pain conditions such as osteoarthritis, neuropathic pain, and other non-cancer chronic pain. However, further research is needed about how the cannabinoid system works with the pain pathway. Using the fact that medicinal cannabis is effective in the treatment of chronic pain and certain rheumatic diseases, in this review, we aim to analyze the role of the cannabinoid system in fibromyalgia syndrome.

We followed Preferred Reporting Items for Systematic Review and Meta-Analysis (PRISMA) guidelines in searching PubMed, MEDLINE (through PubMed), PubMed Central, and Google Scholar using keywords "fibromyalgia, chronic pain, cannabis, cannabinoids, pharmacotherapy, alternative therapy" and Medical Subject Heading (MeSH) words.

After applying inclusion/exclusion criteria and checking for the quality assessment, 22 articles were retrieved and used for the analysis of the role of cannabis in the treatment of fibromyalgia. The two main compounds of cannabis with analgesic and anti-inflammatory properties are cannabidiol (CBD) and delta-9-tetrahydrocannabinol (THC), and their ratio determines the effect on various symptoms of FMS. We included studies regarding the use of cannabinoids in the treatment of fibromyalgia, investigating the use of nabilone, dronabinol (a synthetic analog of THC), Bedrocan (22.4 mg THC, <1 mg CBD), Bediol (13.4 mg THC, 17.8 mg CBD), and Bedrolite (18.4 mg CBD, <1 mg THC).

In the era of the coronavirus disease 2019 (COVID-19) pandemic and opioid crisis, many adverse outcomes are observed in the patients suffering from FMS due to lack of any definitive treatment and promising outcomes from the known treatment options, which led to the need for effective and safer treatment alternatives.

Although the studies reviewed in this article suggest that medical cannabis is a safe and effective treatment for fibromyalgia pain, several limitations regarding dosage, length of treatment, adverse effects, long-term follow-up, and dependence needs further investigation.

## Introduction and background

Fibromyalgia syndrome (FMS) is a pain disorder with an estimated prevalence of 5-7% in the world, with a mean prevalence among the American and European populations of 4% [1]. It is more common in women, with a female to male ratio of 2:1, and can develop at any age. This disease also co-exists with other rheumatic pathologies. It is estimated that about 20-30% of patients with rheumatic diseases have FMS [2].

The syndrome is characterized by chronic widespread pain, fatigue, and sleep disturbance. The exact pathophysiology is still unknown, but the most accepted pathology is the alteration of central pain pathways, which results in hyperalgesia. There is also evidence that supports the role of mast cells in musculoskeletal pain and central sensitization. The mast cells can mediate the activation of microglia through the production of cytokines like interleukin 1 beta (IL-1B), interleukin 6 (IL-6), and tumor necrosis factor (TNF) alpha. Despite all the known facts, the mechanism that links different pathological features including stress, central sensitization, and dysregulation of innate and adaptive immune response is still unknown, making the treatment approach more challenging [3].

A recent study also showed a link between autoantibodies and FMS, as one-third of FMS patients with xerostomia tested positive for Sjogren's syndrome biomarkers and the majority of them were positive for one or more tissue-specific autoantibodies. The diagnosis of FMS is also challenging due to the coexistence of other conditions. FMS is rarely a stand-alone diagnosis, as most patients meet the criteria of other overlapping chronic pain conditions or mental disorders. Once diagnosed, the treatment is also challenging. Various treatment options are available including memantine, naltrexone, tapentadol, duloxetine, palmitoylethanolamide tablets, and cannabinoids. But none of them have 100% promising results [4].

Formulations using cannabis have been used in clinical settings to study its efficacy in reducing pain when traditional options have failed [5]. Cannabinoids may be useful in the management of rheumatic disorders for many reasons, their anti-inflammatory and immunomodulatory activity, and their effect on pain-associated symptoms [4]. The analgesic effect of cannabinoids is primarily mediated by cannabinoid receptors via inhibition of presynaptic gamma-aminobutyric acid (GABA) and glutamatergic transmission [6]. There are two cannabinoid receptors (CB), CB1 and CB2, that are found in the human body. CB1 receptors are predominantly expressed in CNS and CB2 receptors are found mostly outside the CNS. Moreover, activation of these receptors is believed to have anti-nociceptive effects in controlling the human perception of pain. Studies have also shown, these receptors play an important anti-inflammatory role in chronic pain conditions [7].

Finally, despite considerable uncertainty regarding the mechanism of action and its exact role in the management of pain and non-pain symptoms of fibromyalgia, medical cannabis has become a very important focus for research and controversies in the last few years but it also represents hope for many patients [2]. In this systematic review, our main goal is to explore the beneficial therapeutic effects of medicinal cannabis, in addition to its characteristics and its role in the treatment of fibromyalgia.

## Review

Methods

The Preferred Reporting Items for Systematic Review and Meta-Analysis (PRISMA) was used for this study [8]. We identified the articles related to our research question from the databases PubMed, PubMed Central (PMC), MEDLINE (through PubMed), and Google scholar for our research. A search was conducted on April 23, 2021, and an advanced search strategy was used while searching the articles in the main databases. Two reviewers, HK and IQ, went through the screening process and quality assessment independently. Initially, 16,323 articles were found, and when the search was narrowed to the last five years, 3,162 articles were retrieved. Specific inclusion/exclusion criteria were applied and only studies in the English language, with human subjects, randomized clinical trials, observational studies, review, systematic review, and meta-analysis were included and 454 articles were retrieved. After the initial search, 998 articles were retrieved from Google Scholar. We checked for duplicates and 20 duplicates were found and removed. After applying the inclusion/exclusion criteria, the articles that were irrelevant to the research question were excluded.

Results

**Figure 1:**
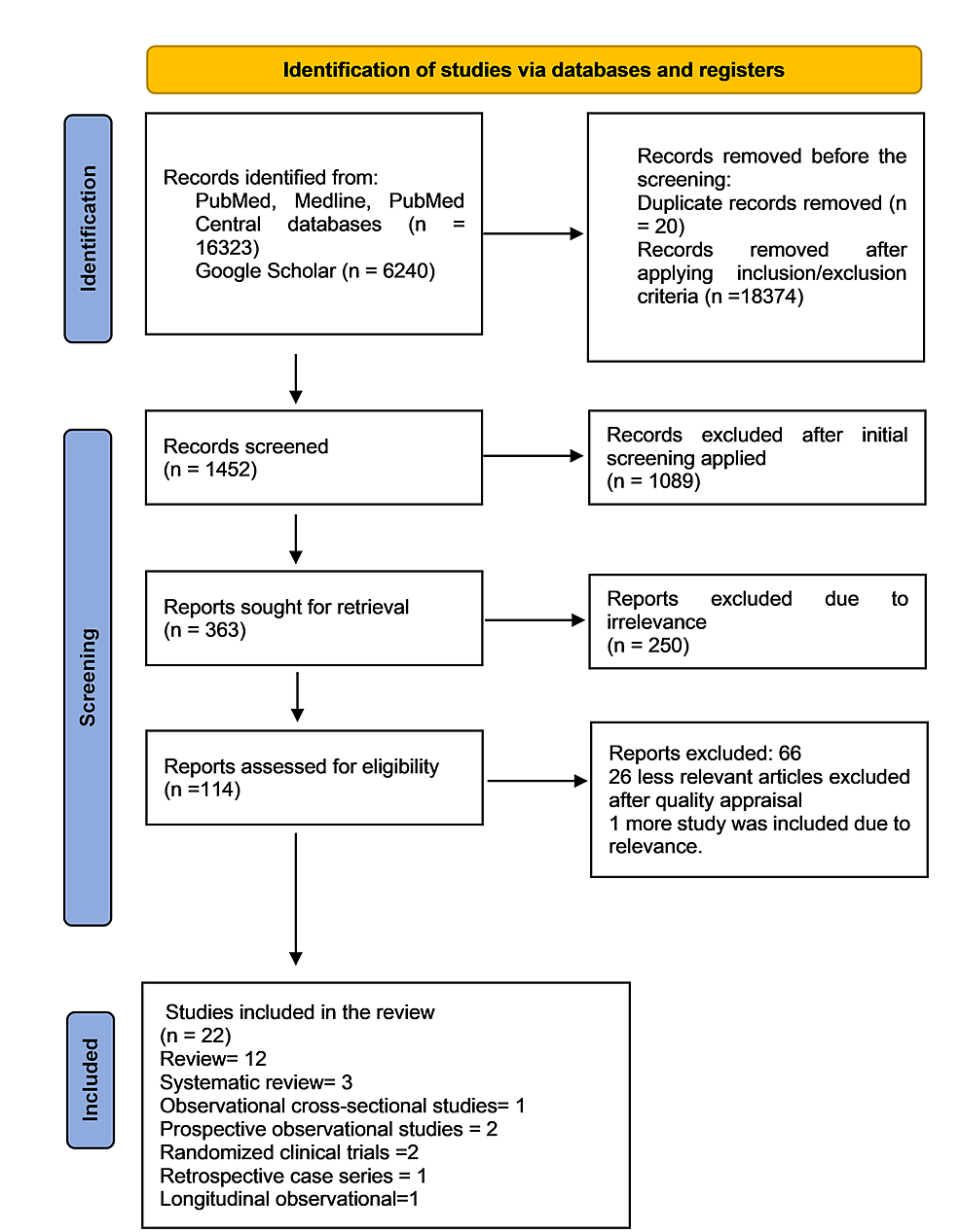
PRISMA flow diagram (2020) showing the search results and selection process. PRISMA, Preferred Reporting Items for Systematic Reviews and Meta-Analysis.

A total of 363 articles were retrieved after title screening. A total of 250 less relevant articles were excluded, and 163 articles were retained. A total of 114 more articles were excluded after checking the eligibility criteria and 49 articles were retrieved and checked for the quality assessment using AMSTAR (assessing the methodological quality of systematic reviews) for systematic review and meta-analysis, Cochrane risk bias assessment tool for clinical trials, Newcastle-Ottawa Scale for observational studies, and SANRA (scale for the quality assessment of narrative review articles) for review articles, and 49 final full-text articles were retrieved for study. A total of 25 more articles were excluded after reading the full text, assessing the quality appraisal and relevance related to the research question. Finally, 22 articles were retrieved for study. Figure 1 shows the flow diagram for the search strategy outlining our search process [8].

Discussion

Fibromyalgia (FM) is a syndrome characterized by chronic pain with multiple tender points, increased pain sensitivity, and other systemic symptoms like cognitive dysfunction, sleep disturbance, anxiety, chronic fatigue, and depression, in the absence of any well-defined underlying organic disease [9]. In most cases, the pain is not explained by inflammation or injury. In other words, there is sensory hyperresponsiveness and hypersensitization to pain [3].

With the recent coronavirus disease 2019 (COVID-19) pandemic situation, the patients with FM syndrome reported adverse mental and physical outcomes, and this exacerbation of symptoms can be related to the increasing level of anxiety, economic hardships, and social isolation [10], which led to the importance of exploring the definitive effective treatment options for FMS.

Pathophysiology and Diagnosis

**Figure 2:**
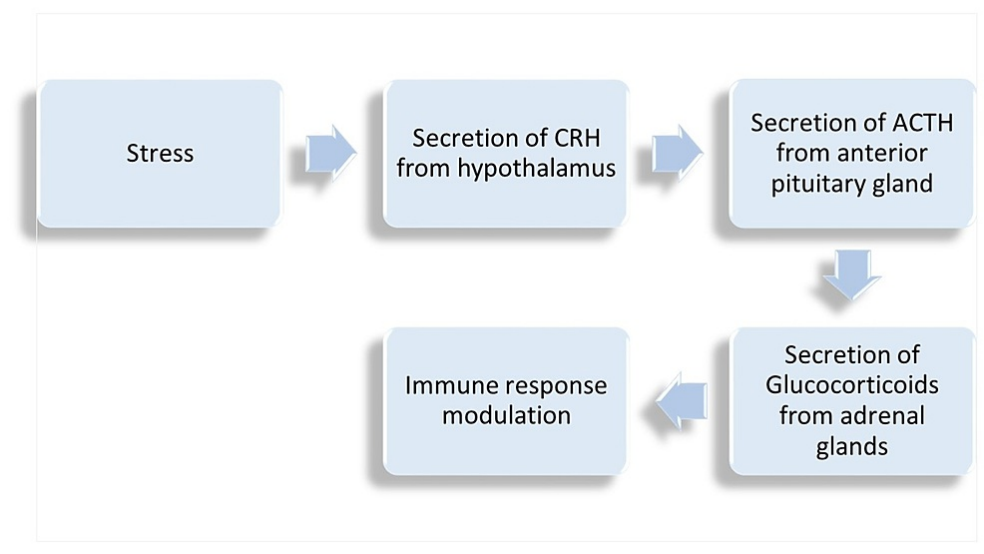
FMS pathogenesis. FMS, fibromyalgia syndrome; CRH, corticotropin-releasing hormone; ACTH, adrenocorticotropic hormone.

The etiology of this disease is still unknown, and the research in this field has expanded considerably, exploring the genetics, immune system, autonomic system, inflammatory response, neurotransmitters, and psychological factors [2]. Initially, it was recognized as pain syndrome in individuals with a high level of stress, but now it is known that there is not a single trigger defining this disease. The most important pathological mechanism is the alteration of central pathways or central sensitization with amplification of pain perception. The hypothalamic-pituitary-adrenal (HPA) axis is considered to play an important role in the establishment of central sensitization [3,4]. Additionally, stress causes the release of corticotropin-releasing hormone (CRH) from the hypothalamus, which acts on the anterior pituitary resulting in the release of adrenocorticotropic hormone (ACTH) from the anterior pituitary, which modulates the immune response through the secretion of glucocorticoids by stimulating the adrenal glands. This includes mast cells degranulation, which can lead to sensitization of peripheral and central nociceptors and the increase of pro-inflammatory cytokines [3]. Figure 2 shows the proposed mechanisms of FMS pathogenesis.

In addition to the stress regulation and inflammatory response, the HPA axis and the sympathetic adrenal-medullary axis are also involved. Some studies in animals also suggest the involvement of T-cells autoimmune response in hyperalgesia; nevertheless, the results of the studies indicating the changes specific to T-cell are inconclusive [3]. One interesting hypothesis favors the role of the thalamic mast cell that may contribute to the release of histamine, interleukin-1 beta, IL-6, tumor necrosis factor (TNF), and calcitonin gene-related peptide, which stimulates nociceptive neurons directly or indirectly by stimulation of microglia [4]. The evidence that supports the role of mast cells in fibromyalgia states that CCL1 (eotaxin-1) and CCL2 (eotaxin-2), which function as potent chemoattractants for inflammatory cells, were found to be elevated in patients with FMS [3].

It is also hypothesized that lack of endocannabinoids activity is the possible pathophysiology of fibromyalgia [1,9,11], and cannabinoids can reduce sensitization of nociceptive sensory pathways in chronic pain states, but there is no evidence enough to support this hypothesis yet [6]. Recent research said FMS pain is non-nociceptive and non-neuropathic, and the new term introduced is “Nociplastic Pain,” referring to the pain without any obvious tissue damage. For example, pain arising from altered nociception despite any clear evidence of inflammation [4]. The term nociplastic pain also has its limitations; it can be applied to some of the pathologies related to FBS but it does not apply to FM syndrome if we consider the bio-psycho-social model to understand the natural history of fibromyalgia [2].

The exact diagnosis requires certain guidelines that reflect not only the classification criteria but also explain the pathogenesis. Due to still unknown pathophysiology, the diagnosis is even more challenging and about 75% of patients remain undiagnosed [4,9]. As it is not possible to rely on a single symptom, various composite indices have been described, encompassing the main features of this poly-symptomatic disease such as pain, fatigue, sleep alteration, neurocognitive disorders, anxiety, and depression. The most widely used diagnostic tools include the Fibromyalgia Impact Questionnaire (FIQ) and its revised version (FIQR), the Fibromyalgia Assessment Status (FAS), the Fibromyalgia Survey Criteria (FSC), and the Patient Health Questionnaire 15 (PHQ15) [2].

Treatment Strategies

Various pharmacological and non-pharmacological therapies can be used for the treatment of FM. Pharmacological therapy is primarily aimed at lowering the level of pro-nociceptive neurotransmitters. There are only a few drugs approved by the Food and Drug Administration (FDA) for FM: pregabalin, duloxetine, and milnacipran [11]. As there is no conclusive evidence providing the benefit of any specific therapy for the treatment of FMS, thus none of the drugs has been approved by the European Medicine Agency [9], and European League against Rheumatism [EULAR] guidelines suggest non-pharmacological interventions as the first line of treatment [12].

Due to the complex nature of the disease, a multidimensional approach is proposed, including non-pharmacological methods in addition to pharmacological treatment, which include acupuncture, cognitive behavioral therapy, hyperbaric oxygen therapy, mindfulness, massage, and transcranial magnetic stimulation. Although outcomes are encouraging, further studies are required to assess the effectiveness of these methods alone or in combination [13].

New treatment options are under investigation: mirtazapine, an alpha-2 antagonist with serotonergic and noradrenergic effects, milnacipran, a serotonin-norepinephrine reuptake inhibitor, and opioids. The mentioned treatments have been assessed for effectiveness, but the results are so far controversial. The cannabis plant seems to be a promising tool to fight fibromyalgia chronic pain [2]. Therefore, there is an intense need to explore other pharmacological effects, efficacy, and safety of cannabis for the treatment of fibromyalgia.

Cannabinoids for Fibromyalgia

Given the fact that we are in the era COVID-19 pandemic and an ongoing opioid crisis, there is an absolute need for effective and safer treatment alternatives for chronic pain syndrome including FM. With a high margin of safety and proposed regulatory effects of tetrahydrocannabinol (THC) and cannabidiol (CBD) on major endogenous pain circuitry systems, cannabis is emerging as a promising alternative treatment option for the management of chronic pain [14].

Regarding the complex function of the endocannabinoid system in pain modulation, it is hypothesized that lack of endocannabinoids activity is among the underlying pathophysiology of FM but there is no clear evidence to support this assumption [1,9,11,15]. It is also hypothesized that cannabinoids reduce the sensitization of nociceptive sensory pathways in chronic pain states [15]. Moreover, the endocannabinoid system is involved in the modulation of other physiological functions, such as inflammation, endocrine function, cognition, memory, nausea, anti-nociception, and vomiting [9]. A study suggests that cannabinoids might function to reduce stress and modulate cognitive and emotional functions [15]. The endocannabinoids act as ligands at cannabinoid receptors CB1 and CB2; CB1 receptors are predominantly expressed in the central nervous system (CNS), whereas CB2 receptors are found mostly outside the CNS. Abundant preclinical data support that when these receptors are activated, pain stimulus is suppressed, influencing nociception [7,15].

The analgesic effects of cannabinoids and their ligands are primarily mediated by the CB1 receptor via inhibition of presynaptic gamma-aminobutyric acid (GABA) and glutamatergic transmission, which suppresses neuronal excitability [6]. Although many cannabinoids are identified, out of them only tetrahydrocannabinol (THC) and cannabidiol (CBD) are the clinically relevant components. Both act on CB1 and CB2 receptors. THC influences pain, appetite, orientation, and mood; while CBD has anti-inflammatory, anti-anxiety, and analgesic effects. Although THC and CBD both act on cannabinoid CB1 and CB2 receptors; THC is a receptor partial agonist, while CBD is a negative allosteric modulator of the CB1 receptor. Due to their varying properties, the proportion of THC to CBD in cannabis products determines the therapeutic and adverse effects [1]. According to the “entourage theory,” the combination of THC and CBD creates a synergistic effect suggesting that there could be a benefit in using cannabis as an analgesic or therapeutic agent [12]. As cannabis and cannabinoids were recommended for the treatment of neuropathic pain and due to the similarities between neuropathic pain and fibromyalgia; it is hypothesized that cannabis or cannabinoids might be effective for fibromyalgia-associated pain as well [12]. Several review articles were assessed for data extraction. The summary of these articles along with the results and conclusions is given in Table 1.

**Table 1:** Showing the summary of systematic review and literature review articles along with results and conclusions. THC, tetrahydrocannabinol; CBD, cannabidiol; FM, fibromyalgia; RCT, randomized control trials.

Authors and year of publication	Purpose of study	Results	Conclusion
Walitt et al. (2016) [15]	To determine the efficacy, safety, and tolerability of cannabinoids for fibromyalgia.	No evidence of at least 30-50% reduction in pain, no significant difference from the effect of placebo.	The tolerability of nabilone is low and no convincing evidence was found suggesting that nabilone can be used for the treatment of FM.
Lawson et al. (2017) [13]	To explore the latest pharmacological strategies for the treatment of fibromyalgia.	The cannabinoids nabilone (0.5-1.0 mg/d) and dronabinol (a synthetic form of delta-9 tetrahydrocannabinol (THC); 7.5 mg/d) significantly reduced pain, depression, and anxiety levels in patients with FM also leading to an improved quality of life.	Due to certain limitations including the incidence of adverse effects and drop-out rates up to 25% during the clinical trials; no concluding evidence can be drawn of cannabinoids for the treatment of FM.
Banerjee et al. (2019) [16]	Medical cannabis for the treatment of chronic pain, neuropathic pain, rheumatologic pain, fibromyalgia.	There are some suggestions of benefit with cannabis for neuropathic pain; however, findings are inconsistent for the effect of cannabis-based medicines in patients with rheumatic disease and fibromyalgia.	The results are inconclusive, cannabis-based medicines may or may not be offered; depending on the type of cannabis-based medicine and patient condition
Cameron et al. (2020) [1]	To study the literature (2015–2019) on the safety and efficacy of medical cannabis for the treatment of fibromyalgia.	Medical cannabis is found to be a safe and effective treatment for fibromyalgia pain.	There are certain limitations in the studies due to which conclusions regarding the use of cannabinoids for pain management in fibromyalgia patients cannot be made.
Tal Gonen et al. (2020) [12]	Use of cannabinoids and cannabis as a treatment for rheumatic diseases.	Cannabis and cannabinoids could relieve some of the symptoms associated with fibromyalgia.	The results are inconclusive of cannabis-based medicines regarding the treatment of FM and need more research.
Tzadok et al. (2020) [11]	To study current and emerging pharmacotherapy for fibromyalgia.	Nabilone and dronabinol showed Improvement in pain and anxiety in several randomized controlled trials and meta-analyses. THC and CBD, therefore, determines the overall effect.	Research suggests the use of cannabinoids for FM patients with sleep abnormalities. Further studies are needed to determine the exact pathogenesis of FM, and endocannabinoid system alteration.
Maffei et al. (2020) [9]	To study the diagnostic criteria for FM as well as to explore pharmacotherapy.	The synthetic cannabinoid and nabilone are superior to placebo and showed significant reductions in the visual analog scale (VAS) for pain.	Some adverse effects were experienced, which suggests further studies to modify the dose-effect relationship.
Bazzichi et al. (2020) [4].	To study the etiology, pathogenesis, and treatment of FM.	There is a promising analgesic role of cannabis products when used alone or in combination for the treatment of fibromyalgia patients.	There are promising analgesic effects of cannabinoids observed in FM patients. The complex behavior of inhaled cannabinoids in patients suffering from chronic pain needs further study.
Birks et al. (2021) [17]	To review the literature regarding the effects of cannabinoids and/or cannabis on chronic pain.	There was no evidence of cannabinoids and/or cannabis effectiveness in individuals suffering from chronic pain.	There was no evidence for the use of cannabinoids and/or cannabis in the treatment of chronic pain. Future studies should be done.
Chang et al. (2021) [18]	Medical cannabis for patients with chronic noncancerous pain including neuropathic pain, low back pain, rheumatoid arthritis, and fibromyalgia.	Cannabinoids can be used for neuropathic pain but first-line therapy should not be replaced. This study was on neuropathic pain including FM, not exclusively on FM; and then results can be inconclusive.	Well-designed and large RCTs with reasonable long-term follow-up are required with detailed discussions on benefits in reducing pain and potential adverse effects are required before its prescription.

The results of several old studies showed certain limitations as most of them involved the use of nabilone and dronabinol (a synthetic delta-9-THC), and concluded with the certain incidence of noticeable adverse effects and low tolerability depending upon the patient’s conditions and loss of follow-up with most of the patients [13,15,16]. However, some recent studies showed that cannabinoids could be safe, effective, and potentially alleviate some of the symptoms associated with fibromyalgia; also, they found that nabilone is superior to placebo and showed significant reductions in visual analog scale (VAS) for pain [1,4,9,12]. One study consisting of cannabinoids for non-cancer pain, which also included FM patients, concluded that cannabinoids can be used for neuropathic pain but should not replace the first-line therapy. As this study was on neuropathic pain including FM, not exclusively on FM, results are inconclusive and we cannot generalize the results based on these findings only [18].

In one other recent study, nabilone and dronabinol showed improvement in pain and anxiety in several randomized controlled trials and meta-analyses. Tetrahydrocannabinol to cannabidiol ratio (THC:CBD) therefore determines the overall effect. It also concluded that manipulating the endocannabinoid system is gradually emerging as another fascinating strategy for treating pain and suggested future research into the clinical utility of endocannabinoid metabolism manipulation in FMS [11].

Therapeutic Use of Cannabis for Fibromyalgia

Findings regarding the use of cannabinoids in the treatment of fibromyalgia consisted of several studies, investigating the use of nabilone, dronabinol, a synthetic analog of THC, Bedrocan (22.4 mg THC, <1 mg CBD), Bediol (13.4 mg THC, 17.8 mg CBD), and Bedrolite (18.4 mg CBD, <1 mg THC) [19]. The clinical studies assessed and their findings are summarized in Table 2.

**Table 2:** Showing the observational studies, clinical trials, and case series conducted on the use of cannabis for the treatment of fibromyalgia. THC, tetrahydrocannabinol; CBD, cannabidiol; FM, fibromyalgia; FIQ, Fibromyalgia Impact Questionnaire; NRS, Numerical Rating Scale; ODI, Oswestry Disability Index; WPI, Wide Pain Index; SyS, severity score.

Author and year of publication	Purpose of study	No. of patients	Type of study	Results	Conclusion
Yassin et al. (2019) [20]	Effect of adding cannabis to analgesic treatment in FM patients with low back pain.	31	Observational cross-over single-center study.	Medical cannabis showed a significant improvement in three months after initiation of therapy and the improvement was maintained at six months.	This observational crossover study showed improvement of back pain in FM patients treated with medical cannabis. Further randomized clinical trial studies are suggested for assessment.
Van de Donk et al. (2019) [19]	The analgesic effects of pharmaceutical-grade cannabis in chronic pain patients with fibromyalgia.	20	Randomized placebo-controlled crossover trial.	This experimental trial showed the complex behavior of different inhaled cannabinoids compounds in chronic pain patients with just small analgesic responses after a single inhalation.	Further studies are needed to determine long-term treatment effects on spontaneous pain scores, THC–CBD interactions, and their role in pain relief.
Sagy et al. (2019) [21]	Safety and efficacy of medical cannabis in fibromyalgia.	367	A prospective observational study.	Pain intensity (scale 0–10) reduced from a median of 9.0 at baseline to 5.0 (p < 0.001), and 81.1% of patients achieved treatment response. Mild adverse effects were dizziness, dry mouth, and gastrointestinal symptoms.	Medical cannabis appears to be a safe and effective alternative for the treatment of fibromyalgia symptoms. Standardization of treatment regimens is required.
Giorgi et al. (2020) [22]	Adding medical cannabis to standard analgesic treatment for fibromyalgia.	102	A prospective observational study.	After six months, 50% showed a moderate improvement in the anxiety and depression scales. One-third experienced mild adverse events but did not cause any significant treatment modifications.	There is a possible clinical advantage of medical cannabis in FM patients, especially in those with sleep dysfunctions; further studies are needed to confirm these data.
Chaves et al. (2020) [23]	Ingestion of THC-rich cannabis oil in people with fibromyalgia.	17	A randomized, double-blind, placebo-controlled clinical trial.	Cannabis showed a decrease in Fibromyalgia Impact Questionnaire (FIQ) score in comparison with the placebo group (p = 0.005). There were no intolerable adverse effects.	Cannabinoids can be used to reduce symptoms and increase the quality of life of patients with fibromyalgia. Future studies are still needed to assess long-term benefits.
Safakish et al. (2020) [14]	Medical cannabis for pain management and quality of life improvement.	751	A longitudinal, prospective, observational study.	Medical cannabis was associated with improvements in pain severity and interference (p < 0.001) observed at one month and maintained over 12 months.	The results were promising but the percentage of patients with fibromyalgia included in this study is 17.6%, which is very low to make any conclusion.
Mazza et al. (2021) [24]	Medical cannabis for the treatment of fibromyalgia syndrome.	38	A retrospective, open-label case series.	Significant improvements (p < 0.01) were observed in NRS, ODI, WPI, and SyS for 12 months.	Cannabinoids may be used as an alternative treatment for patients with FM who are unresponsive to conventional therapy. However, it is limited by the incidence of non-serious adverse effects.

In one experimental study designed to examine the effect of adding medical cannabis to analgesic treatment, which consisted of 38 patients treated for three months with standard analgesic therapy with minor improvement in the symptoms, treated with medical cannabis therapy for a minimum of six months, which resulted in higher improvement in all patient-reported outcomes (PROs), which included Fibromyalgia Impact Questionnaire (FIQ), visual analog scale (VAS), Oswestry Disability Index (ODI), and lumbar range of motion (ROM), which was recorded using the modified Schober’s test [20].

Another study, consisting of 20 patients carried on the same principle but different cannabis compounds, showed the complex behavior of different inhaled cannabinoids compound in chronic pain patients with just small analgesic responses after a single inhalation. Four different cannabis varieties were tested including Bedrocan (22.4 mg THC, <1 mg CBD), Bediol (13.4 mg THC, 17.8 mg CBD), Bedrolite (18.4 mg CBD, <1 mg THC), and a placebo variety without any THC or CBD. The study results showed that none of the treatments had an effect greater than placebo on spontaneous or electrical pain responses, although more subjects receiving Bediol displayed a 30% decrease in pain scores compared to placebo. It also showed antagonistic pharmacodynamic interactions of THC and CBD. So further studies are needed to determine long-term treatment effects on spontaneous pain scores, THC-CBD interactions, and their effects on pain relief. In this study, two experimental pain tests were performed, the electrical pain test and pressure pain test; pressure pain threshold increased significantly in patients treated with Bedrocan and Bediol. In addition, Bediol had notably greater effects than Bedrolite so significantly more patients responded to Bediol. Bedrolite, a cannabis variety with a high CBD content, was devoid of analgesic activity in any of the spontaneous or evoked pain models [19].

About further studies, one study consisting of 367 patients conducted to investigate the safety and efficacy of medical cannabis in fibromyalgia was conducted on patients who were willing to answer the questionnaire in a specialized medical cannabis clinic between 2015 and 2017. It concluded that medical cannabis appears to be a safe and effective alternative for the treatment of fibromyalgia symptoms with certain limitations like standardization of treatment compounds and regimens, which require more research. This study included patients with six months follow-up and the response rate was 70.8%. The pain intensity reduced from a median of 9.0 at baseline to 5.0 on a pain scale 0-10 (p < 0.001), and 194 patients (81.1%) achieved treatment response. The most common adverse effects were mild and included dizziness, dry mouth, and gastrointestinal symptoms [21].

With previous knowledge on two different compounds (Bedrocan and Bediol), another study carried out to study further outcomes, included 102 FM patients to assess any clinical improvement following the addition of medical cannabis treatment (MCT) to the stable (≥ three months) standard an­algesic treatment of FM patients. Patients were prescribed two oil-diluted cannabis extracts: Bedrocan (22% THC, <1% CBD), and Bediol (6.3% THC, 8% CBD). FM severity was periodically assessed using the Fibro­myalgia Impact Questionnaire (FIQ), Fibromyalgia Assessment Scale (FAS), Functional Assessment of Chronic Illness Therapy (FACIT) Fatigue score, Pittsburgh Sleep Quality Index (PSQI), and Zung De­pression and Anxiety Scales. During the study, patients were allowed to reduce or stop their concomitant analgesic therapy. Finally, 50% showed a moderate improvement in anxiety and depression; besides, analgesic treatment was reduced or suspended in 47% of the patients. In general, only one-third ex­perienced mild adverse events. Overall, it showed that adjunctive MCT offers a possible clinical advantage in FM pa­tients [22].

In another study, a double-blind randomized placebo-controlled clinical trial consisting of 17 women that were conducted for eight weeks to determine the benefit of THC-rich cannabis oil on symptoms and quality of life, concluded that cannabinoids can be a low-cost and well-tolerated therapy to reduce symptoms and increase the quality of life of patients with fibromyalgia. The Fibromyalgia Impact Questionnaire (FIQ) was applied at pre- and post-intervention moments and in five visits over eight weeks. After the intervention, the cannabis group presented a significant decrease in FIQ score in comparison with the placebo group [23].

A study on the effect of cannabinoids on chronic pain patients including 132 FM patients out of a total of 751 patients with chronic pain with other underlying conditions, also showed promising results, but the percentage of patients with fibromyalgia included in this study is 17.6%, which is very low to make a relevant assumption. Nevertheless, results concluded over patients were promising with improved pain scores over 12 months. Moreover, the medical cannabis (MC) treatment course in this study was not associated with increases in the frequency of undesired adverse events, but rather decreased the frequency of headaches, fatigue, feelings of anxiety, and nausea [14].

Recently a retrospective, open-label case series consisting of 38 patients was conducted to study the efficacy and adverse events (AEs) of short- and long-term MC treatment for FM concluded that MC may be used as an alternative treatment for patients with FMS who are unresponsive to conventional therapy. However, its application may be limited by the incidence of non-serious adverse effects. The study was conducted for 12 months with follow-up at 1, 3, and 12 months. The results were interpreted based on certain scales including Numerical Rating Scale (NRS), Oswestry Disability Index (ODI), Hospital Anxiety and Depression Scale (HADS), Widespread Pain Index (WPI), and Severity Score (SYS). The most common side effects were mental confusion, dizziness, nausea/vomiting, and restlessness/irritation [24].

All these studies showed the significant advantage of MC in treating pain in patients with FMS with a few non-serious adverse effects. Medical cannabis appears to be a safe and effective alternative for the treatment of fibromyalgia symptoms.

As FM is a syndrome of symptoms with still not completely known pathogenesis, it might pose additional difficulty in treating it. Also, with the recent advancements and studies regarding lack of endocannabinoid activity as a possible cause of the disease process, cannabis is considered a future hope for treating FM syndrome as it has shown a significant advantage in treating this condition with very few adverse effects. Future studies are still needed to assess long-term benefits, THC-CBD interactions, and their effects on pain relief, to determine and standardize treatment regimens, to assess long-term benefits, dose-response relationship, and dependence.

Limitations

There are certain limitations in our study, as we only used articles in the English language, conducted only on humans, and published in the last five years; hence, certain valuable studies could have been excluded.

## Conclusions

Our main aim was to assess the safety and efficacy of cannabinoid compounds for the treatment of FMS. At this point, the data suggest that the use of cannabinoids and cannabis carries limited side effects in the treatment of FM, and they can also improve some common and debilitating symptoms associated with FM, thus making them an adequate potential treatment option, when other treatment lines have been exhausted.

Ultimately, we believe that the use of cannabis and cannabinoids for pain relief in fibromyalgia has shown great potential and maybe a source of hope for those suffering from chronic pain associated with this condition, and for the physicians treating them; however, benefits need to be weighed against the harmful effects, and more research into this area should be conducted, for longer periods, to assess for long-term efficacy, adverse effects, and dependence. The ratio of TCH:CBD also seems to be an important factor in the outcome, which needs further research. So more clinical trials with long-term follow-up and study on the dose-response relationship and dependence need to be done.
